# Longitudinal Evaluation of Research Career Intentions Among US Medical Students

**DOI:** 10.1001/jamanetworkopen.2026.11430

**Published:** 2026-05-12

**Authors:** Alexandra M. Hajduk, Meghan O’Connell, Allison Aviles, Jeph Herrin, Mytien Nguyen, Shruthi Venkataraman, Marney A. White, John Paul Sánchez, Rachel K. Wolfson, Dowin Boatright, Sarwat I. Chaudhry

**Affiliations:** 1 Department of Internal Medicine, Yale School of Medicine, New Haven, Connecticut; 2 Department of Immunobiology, Yale School of Medicine, New Haven, Connecticut; 3 Ronald O. Perelman Department of Emergency Medicine, NYU Grossman School of Medicine, New York, New York; 4 Department of Social and Behavioral Sciences, Yale School of Public Health, New Haven, Connecticut; 5 Albert Einstein College of Medicine, Bronx, New York; 6 Pritzker School of Medicine, University of Chicago, Chicago, Illinois

## Abstract

**Importance:**

The physician-scientist workforce has been in decline for decades, and the shortage of physician-scientists from racial and ethnic groups that are underrepresented in medicine (URiM) is particularly acute. Medical school is a key developmental period during which research career intentions (RCI) may change, yet little is known about RCI evolution during this period.

**Objective:**

To evaluate factors associated with RCI among first-year medical students, overall and by URiM status.

**Design, Setting, and Participants:**

This is a cross-sectional analysis of baseline data from the ongoing Longitudinal Evaluation of Research Career Intentions Among Medical Students Study. Participants were first-year medical students enrolled during the 2024 to 2025 academic year at US medical schools accredited by the Liaison Committee on Medical Education. MD-PhD students were excluded.

**Exposures:**

The baseline survey collected information on sociodemographic factors, pre–medical school and first-year research experiences (eg, research education, research participation, mentorship, and authorship), medical school learning environment, and psychosocial characteristics.

**Main Outcomes and Measures:**

The primary outcome was RCI in year 1 of medical school, defined as intending to be significantly or exclusively involved in research during one’s medical career. Characteristics associated with RCI were evaluated using stability selection.

**Results:**

Among 1136 first-year medical student respondents (mean [SD] age, 24.7 [2.7] years; 790 female [69.5%]) at 134 US medical schools, 26.9% (306 students) reported RCI, with a slightly higher prevalence among URiM students than non-URiM students (246 students [28.2%] vs 60 students [23.9%]; standardized mean difference, 0.097). Prematriculation factors associated with RCI among all students included research participation (odds ratio [OR], 1.51; 95% CI, 1.13-2.00), conference presentation (OR, 1.56; 95% CI, 1.16-2.09), and manuscript authorship (OR, 1.38; 95% CI, 1.03-1.86). Postmatriculation factors included research participation (OR, 1.74; 95% CI, 1.31-2.30) and having a physician-scientist role model (OR, 1.72; 95% CI, 1.31-2.30). Factors uniquely associated with RCI among URiM students included prematriculation research experiences (OR, 1.51; 95% CI, 1.14-2.01) and presentation of research (OR, 1.57; 95% CI, 1.17-2.11) and postmatriculation manuscript authorship (OR, 1.84; 95% CI, 1.04-3.25). Among non-URiM students, only postmatriculation factors were uniquely associated with RCI, including having a research mentor (OR, 1.76; 95% CI, 1.34-2.31) and receiving education about physician-scientist work-life balance (OR, 1.63; 95% CI, 1.24-2.15).

**Conclusions and Relevance:**

This cross-sectional analysis of baseline data from an ongoing cohort study found that RCI was prevalent among first-year medical students and was associated with characteristics and experiences prior to matriculation and during year 1. Some factors associated with RCI differed between URiM and non-URiM medical students, suggesting distinct pipelines to research career development. These findings highlight opportunities to support physician-scientist training through tailored education, exposure, and mentorship.

## Key Points

**Question:**

What are the research career intentions (RCI) of first-year medical students?

**Findings:**

Among 1136 first-year medical students from 134 US medical schools enrolled in this cross-sectional study, 26.9% endorsed RCI in year 1. Factors associated with RCI in year 1 included prior and ongoing research education and participation, as well as having physician-scientist mentors or role models; some factors differed between students from underrepresented in medicine (URiM) groups and non-URiM students.

**Meaning:**

RCI is prevalent among first-year medical students, and factors associated with RCI reveal opportunities to strengthen the physician-scientist pipeline through innovations to admissions, research education, engagement, and mentorship.

## Teaser

This cross-sectional study examines factors associated with research career intentions among first-year medical students, overall and by underrepresented in medicine status.

## Introduction

Physician-scientists (ie, physicians who spend a substantial proportion of their working time contributing to scientific investigation) play a vital role in biomedical research by bringing clinically relevant questions to research and translating discoveries into improved patient care. Yet, the physician-scientist workforce has steadily declined, with physicians reporting research as a primary activity comprising just 1.6% of the physician workforce today, compared with 4.6% in 1985.^1^ The aging of the current physician-scientist workforce^2^ and low interest in research careers among graduating medical students^3^ create concern about the ability of the US to remain a global leader medical research.^4^

The physician-scientist shortage is particularly severe among racial and ethnic groups historically underrepresented in medicine (URiM),^5^ who constitute 33% of the US population^6^ and 25% of recent medical school matriculants^7^ but just 5% of National Institutes of Health (NIH)–funded investigators.^8^ Despite efforts by the NIH and other stakeholders to make the physician-scientist workforce more accurately represent the US population,^9^ little meaningful progress has been made.^10^

Medical school is a critical period for the physician-scientist development. Previous work suggests that more than 60% of medical school graduates’ research career intentions (RCI) change, either increasing or decreasing, during medical school^11,12^ and that RCI at graduation is associated with research careers.^13^ One study^11^ found that URiM students were equally as likely to endorse RCI at matriculation as non-URiM students but more likely to lose interest in a research career by graduation. These findings demonstrate the importance of medical school in shaping RCI and suggest differences in the evolution of RCI for URiM medical students. However, little contemporary data exist about the variability, timing, and determinants of change in RCI among MD students. Prior research has focused mainly on MD-PhD students, a group that is vitally important to the physician-scientist workforce but comprises only 3% of medical students.^14^ Studies of MD (ie, not MD-PhD) students have been secondary analyses of Association of American Medical Colleges (AAMC) data,^11^ which lack granular data on research experiences needed to inform interventions.

Here, we present work from the ongoing Longitudinal Evaluation of Research Career Intentions Among Medical Students (LEAP) study, which aims to characterize trajectories of RCI throughout medical school in a diverse cohort of medical students and to identify factors that can be targeted to improve recruitment and retention of MD students into the physician-scientist pipeline. In this article, we describe the cohort and factors related to RCI during year 1 of medical school.

## Methods

**Figure 1.**
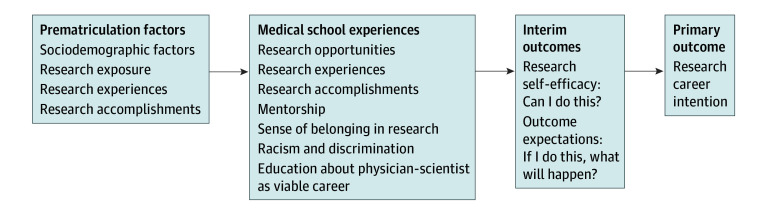
Flowchart of Conceptual Model of Research Career Intention Evolution Among Medical Students

LEAP is an ongoing 5-year prospective study that recruited a nationwide cohort of first-year medical students who will be followed through graduation. Guided by Social Cognitive Career Theory^15^ (Figure 1), we hypothesize that premedical experiences and sociodemographic characteristics, as well as characteristics and experiences of medical school, impact research self-efficacy and outcome expectations, which, in turn, impact RCI. The Yale University institutional review board approved the study. This cross-sectional study followed Strengthening the Reporting of Observational Studies in Epidemiology (STROBE) reporting guidelines.

### Eligibility and Screening

Eligible participants were first-year MD or MD-master’s students enrolled in Liaison Committee on Medical Education–accredited US medical schools during academic year 2024 to 2025. Owing to their notable underrepresentation in the physician-scientist workforce, we oversampled URiM students. We excluded individuals whose medical student status could not be verified and MD-PhD students. We capped per-school enrollment at 30 participants and discontinued enrollment of non-URiM students once recruitment targets were met.

Twelve medical student recruitment champions recruited students through social media, medical student organizations, and peer networks. Recruited students completed an online screener, self-reporting race, ethnicity, and medical school enrollment status. We partnered with AAMC to verify medical school enrollment. Participants were enrolled in the study once they completed informed consent and the baseline survey. Enrolled participants received $75 gift cards.

### Assessments

Details of the annual assessments, which began in January 2024 and will continue through the spring of 2028, are presented in eTable 1 in Supplement 1. Surveys include validated instruments, measures used in prior research, and items developed for this study with guidance from content experts.

### Measures

#### Research Career Intentions

Measures collected during the year 1 survey are described here and in eTable 2 in Supplement 1. We adapted an existing 2-part AAMC Graduation Questionnaire question^16^ to assess RCI to specifically focus on posttraining intentions. Participants were asked, “In which of the following activities do you plan to participate during your career (after completing your training, including residence and fellowship, as applicable)?” with research as one response option. If a participant endorsed research as a planned activity, they were asked, “How extensively do you expect to be involved in research during your medical career?”, with response options of involved in a limited way, significantly involved, and exclusively involved. Participants who responded significantly involved or exclusively involved were characterized as having RCI. This measure has high validity; a 2019 study found that a similar measure of RCI was associated with future receipt of NIH funding.^13^

#### Race, Ethnicity, and URiM Status

We collected self-reported race and ethnicity from respondents via the question, “Which racial and/or ethnic categories describe you? Select all that apply,” with the following options: American Indian or Alaska Native, Asian, Black or African American, Hispanic or Latinx, Middle Eastern or North African, Native Hawaiian or Pacific Islander, White, or other (other race was self-reported, so we did not define this category a priori). We defined URiM status according to the NIH definition^17^: American Indian or Alaska Native, Black or African American, Hispanic or Latinx, Native Hawaiian or Pacific Islander, or multiracial including 1 of the above races and ethnicities. Participants identifying as Asian, Middle Eastern or North African, and/or White only, or multiracial inclusive of Asian, Middle Eastern or Northern African, and White were categorized as non-URiM.

#### Research Experiences Before Medical School

We used questions adapted from the American Medical College Application Service^18^ and the AAMC Matriculating Student Questionnaire^19^ to elicit information on premedical research activities, including research-related pipeline programs, assistantships, internships, and gap years. We also asked participants about research mentorship, authorship, and conference presentations prior to medical school.

#### Research Experiences During Medical School

We developed questions to collect information about research experiences during medical school, including the timing, duration, and types of research conducted; research-related coursework; financial support for student research; motivations for conducting research; and satisfaction with research experiences. We collected information on research skills from a 7-item assessment developed at Stanford.^20^ We also asked about publication authorship, conference presentations, and receiving education about the physician-scientist career path (eg, employment potential, compensation, and work-life balance). We evaluated perceived mentorship quality using the abridged Mentor Competency Assessment^21^ and mentor accessibility with questions from the Medical Student Scholar-Ideal Mentor Scale.^22^

#### Psychosocial Characteristics

We measured symptoms of anxiety and depression with the 4-item Patient Health Questionnaire,^23^ grit with the 8-item Short Grit Scale,^24^ and burnout with the 2-item Maslach Burnout Inventory.^25^ We assessed the size and types of participants’ social networks. We measured quality of life using an AAMC Year Two Questionnaire^26^ question about overall well-being within the past week. We measured imposter syndrome with the Short Clance Impostor Phenomenon Scale.^27^

#### Medical School Environment

We used the 17-item Medical School Learning Environment Survey^28^ to measure students’ perceptions of academic, social, and emotional aspects of the learning environment and the Student Sense of School Belonging Scale^29^ to measure the sense of belonging in medical school. We assessed institutional race climate using the Diversity Engagement Survey^30^ and a subscale of the Race Climate Survey.^31^ We measured discrimination with the Everyday Discrimination Scale^32^ and mistreatment with items from the Graduation Questionnaire.^16^ We developed questions to collect information about URiM representation among medical school leadership and institutional support for URiM students and faculty.

#### Hypothesized Mediators of RCI

We assessed research self-efficacy using the Research Self-Efficacy Scale.^33^ We used the Outcome Expectations subscale developed by Byars-Winston et al^34^ to evaluate outcomes expectations for a research career. The subscale has a score range of 1 to 5, with higher scores indicating higher outcomes expectations.

### Statistical Analysis

For this analysis of baseline data, we first evaluated differences in characteristics between URiM and non-URiM participants via standardized mean difference (SMD),^35,36^ with SMDs greater than or equal to 0.2 (ie, differences ≥0.2 SD between groups) indicating nonnegligible differences.^37^ Given the large number of candidate variables (79 variables) relative to sample size, we first used stability selection^38^ to obtain a parsimonious set of factors, applying a least absolute shrinkage and selection operator with random factor and sample selection to impose controlled error bounds^39,40^; missing data were imputed using random forest imputation.^41^ Stability selection was implemented using 80 000 iterations (approximately 1000 per candidate variable, 10 variables each iteration, 500 subsamples each iteration, and a maximum pairwise family error rate of 1). This algorithm was applied to the full cohort and the subgroups of URiM and non-URiM participants separately; from each application, we retained all variables with maximum peak selection probability greater than 0.50.^38^ In the second stage, we estimated logistic regression models using the retained factors from the first stage. Missing data were managed using 10 imputed datasets. All retained factors were assessed for collinearity using variance inflation factor diagnostics and were then included in a single multivariate logistic regression model for each cohort, with factors with *P* < .10 retained in the final multivariable models. Statistical significance was set at 2-sided *P* < .05. Analyses were performed using R statistical software version 4.5.0 (R Project for Statistical Computing) and Stata statistical software version 19.5 (StataCorp).

## Results

**Figure 2.**
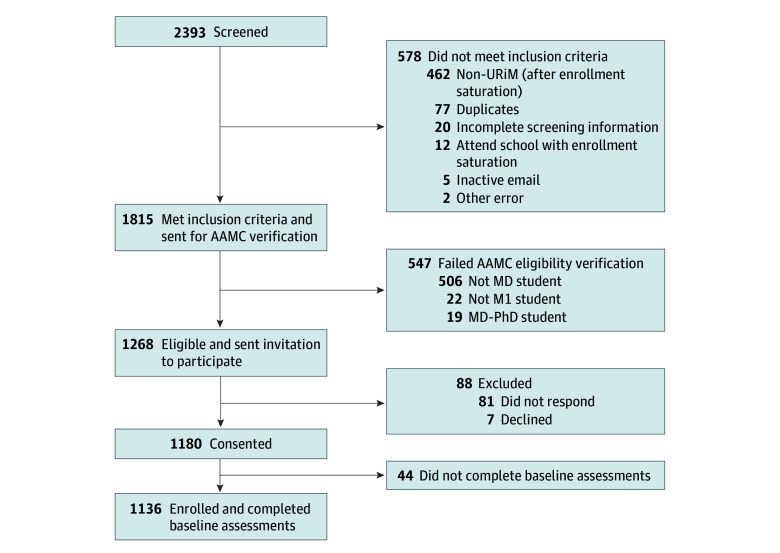
Flowchart of Longitudinal Evaluation of Research Career Intentions Among Medical Students Screening and Enrollment AAMC indicates Association of American Medical Colleges; M1, year 1 of medical school; and URiM, underrepresented in medicine.

From January to May 2024, we screened 2393 students and found 1268 eligible to participate (Figure 2). The most common reason for exclusion was inability to verify medical student status (547 participants). Among those eligible, 1136 students representing 134 US MD-granting programs (of 151 programs nationwide) were enrolled in the study.

Characteristics of enrolled participants are reported in Table 1 and Table 2 (complete description of characteristics is found in eTable 3 in Supplement 1). The mean (SD) age was 24.7 (2.7) years, and 790 students (69.5%) were female. More than three-quarters (883 students [77.8%]) of the sample self-identified as URiM, with nonmutually exclusive race and ethnicity representation as follows: American Indian or Alaska Native (29 students [2.6%]), Asian (162 students [14.3%]), Black or African American (514 students [45.2%]), Hispanic or Latinx (359 students [31.6%]), Native Hawaiian or Pacific Islander (19 students [1.7%]), Middle Eastern or North African (43 students [3.8%]), White (292 students [25.7%]), and other (20 students [1.8%]). Prior to medical school, 932 students (82.2%) reported substantial research experience (eg, undergraduate research pipeline program, research internship or externship, or research gap year), 470 (41.4%) presented research at a conference, and 412 (36.3%) authored a manuscript (Table 1). At the time of the survey, 396 students (34.9%) were conducting research, 526 (46.4%) had identified a research mentor, and 62 (6.0%) had authored a paper during medical school (Table 2). Almost one-quarter (270 students [23.8%]) identified a physician-scientist role model. Reasons for conducting research during medical school were to increase competitiveness for residency (369 students [32.5%]), enhance health equity (112 students [9.9%]), and satisfy curricular requirements (70 students [6.2%]).

**Table 1.** Select Characteristics of Longitudinal Evaluation of Research Career Intentions Among Medical Students Participants According to URiM Status

Characteristic	Participants, No. (%)			SMD
	All (N = 1136)	URiM (n = 883)	Non-URiM (n = 253)	
Sex				
Assigned female at birth	790 (69.5)	617 (69.9)	173 (68.4)	0.059
Assigned male at birth	345 (30.4)	265 (30.0)	80 (31.6)	
Prefer not to answer	1 (0.1)	1 (0.1)	0	
From a disadvantaged background	515 (45.3)	451 (51.1)	64 (25.3)	0.550
Current financial insecurity	526 (46.3)	431 (48.8)	95 (37.5)	0.224
Race and ethnicity (nonexclusive)				
American Indian or Alaska Native	29 (2.6)	29 (3.3)	0	NA
Asian	162 (14.3)	47 (5.3)	115 (45.5)	0.018
Black or African American	514 (45.2)	514 (58.2)	0	NA
Hispanic or Latinx	359 (31.6)	359 (40.7)	0	0.813
Middle Eastern or North African	43 (3.8)	22 (2.5)	21 (8.3)	0.259
Native Hawaiian or Pacific Islander	19 (1.7)	19 (2.2)	0	NA
White	292 (25.7)	156 (17.7)	136 (53.8)	1.039
Other^a^	20 (1.8)	16 (1.8)	4 (1.6)	NA
Prior to medical school				
Substantial research experience^b^	932 (82.2)	705 (80.0)	227 (89.7)	0.273
Presented an oral or poster presentation	470 (41.4)	363 (41.1)	107 (42.3)	0.024
Authored a manuscript	412 (36.3)	304 (34.4)	108 (42.7)	0.170
Student Medical College Admission Test score range				
481-504	319 (28.1)	304 (34.4)	15 (5.9)	1.060
505-509	264 (23.2)	225 (25.5)	39 (15.4)	
510-514	234 (20.6)	169 (19.1)	65 (25.7)	
515-527	258 (22.7)	133 (15.1)	125 (49.4)	
School climate, Medical School Learning Environment Survey score, mean (SD)^c^	3.75 (0.45)	3.73 (0.47)	3.82 (0.39)	0.196

Abbreviations: NA, not applicable; SMD, standardized mean difference; URiM, underrepresented in medicine.

^a^ Other race was self-reported, so we did not define this category a priori.

^b^ Refers to participation in a pre–medical school research pipeline program, paid or unpaid research internship, summer research internship or externship, or a research gap year.

^c^ The score range is 1 to 5, with higher scores indicating more positive perceptions of the medical school learning environment.

**Table 2.** Select Characteristics Related to Research in Year 1 of Medical School of Longitudinal Evaluation of Research Career Intentions Among Medical Students Participants, According to URiM Status

Characteristic	Participants, No. (%)			SMD
	All (N = 1136)	URiM (n = 883)	Non-URiM (n = 253)	
Research scientist career path is positively presented	629 (55.4)	494 (55.9)	135 (53.4)	0.189
Research coursework is required	556 (48.9)	441 (49.9)	115 (45.5)	0.090
Activities during medical school				
Conducts research	396 (34.9)	305 (34.5)	105 (41.5)	0.127
Has a research mentor	526 (46.4)	421 (47.8)	105 (41.5)	0.127
Has a physician-scientist role model	270 (23.8)	212 (24.0)	58 (22.9)	0.029
Medical students are offered financial support for student research	648 (57.0)	495 (56.1)	153 (60.5)	0.087
Proposed mediators of research career intention, mean (SD), scores				
Research self-efficacy^a^	34.11 (5.93)	34.00 (6.02)	34.47 (5.60)	0.033
Outcome Expectations scale^b^	3.51 (0.80)	3.56 (0.81)	3.35 (0.74)	0.263

Abbreviations: SMD, standardized mean difference; URiM, underrepresented in medicine.

^a^ The score range is 11 to 44, with higher scores indicating higher research self-efficacy.

^b^ The score range is 1 to 5, with higher scores indicating greater outcome expectations.

Differences between URiM and non-URiM participants are reported in Table 1, Table 2, and eTable 3 in Supplement 1. URiM participants were older (mean [SD] age, 24.9 [2.8] vs 24.1 [2.2] years; SMD = 0.331) and more likely to come from a socioeconomically disadvantaged background (451 students [51.1%] vs 64 students [25.3%]; SMD = 0.550) compared with non-URiM participants (Table 1). Pre–medical school research experience was lower among URiM than non-URiM students (705 students [80.0%] vs 227 students [89.7%]; SMD = 0.273) (Table 2). URiM and non-URiM students differed in their primary reason for conducting research during medical school, with URiM students being more likely to report to enhance health equity as their primary reason (96 students [10.9%] vs 16 students [6.3%]; SMD = 0.250) (eTable 3 in Supplement 1). URiM and non-URiM students reported similar rates of conference presentations and manuscript authorship prior to medical school and were equally likely to conduct research, have a research mentor, have a physician-scientist role model, and receive instruction in research methods and the physician-scientist career path during year 1. URiM students reported higher outcomes expectations for research (mean [SD] score on the Outcome Expectations subscale of the Research Self-Efficacy Scale, 3.6 [0.8] vs 3.4 [0.7]; SMD = 0.263).

More than one-quarter (306 students [26.9%]) of the first-year medical students reported RCI in year 1; URiM students were slightly more likely than non-URiM students to report RCI (246 students [28.2%] vs 60 students [23.9%]; SMD = 0.097). Characteristics of URiM students and non-URiM students according to year-1 RCI are reported in eTables 4 and 5 in Supplement 1, respectively.

The stability selection process identified 7 factors for the full cohort, 7 factors for the URiM cohort, and 3 factors for the non-URiM cohort. Collinearity assessment with variance inflation factor identified no collinear factors, so all were retained for multivariable selection.

**Figure 3.**
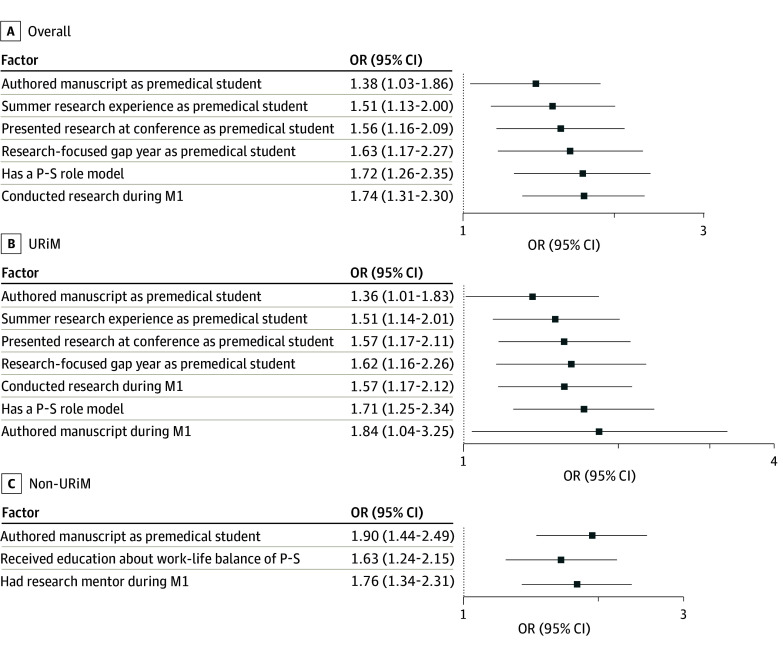
Dot Plot of Factors Associated With Research Career Intentions Among First-Year Medical Students M1 indicates year 1 of medical school; OR, odds ratio; P-S, physician-scientist; and URiM, underrepresented in medicine.

Four prematriculation factors and 3 postmatriculation factors were associated with RCI in year 1 among all students (Figure 3A). Students with substantial prematriculation research experience (ie, conducting summer research) (odds ratio [OR], 1.51; 95% CI, 1.13-2.00) and having a research-focused gap year (OR, 1.63; 95% CI, 1.17-2.27) were more likely to report RCI. Participants who presented an oral or poster presentation at a conference (OR, 1.56; 95% CI, 1.16-2.09) or authored a manuscript (OR, 1.38; 95% CI, 1.03-1.86) prior to medical school were also more likely to report RCI. Once matriculated, participants who conducted research (OR, 1.74; 95% CI, 1.31-2.30) or reported having a physician-scientist role model (OR, 1.72, 95% CI, 1.26-2.35) were more likely to report RCI.

Four prematriculation and 3 postmatriculation factors were associated with RCI in year 1 among URiM students (Figure 3B). URiM students who undertook substantial pre–medical school research experiences, including summer research (OR, 1.51; 95% CI, 1.14-2.01) or a research-focused gap year (OR, 1.62; 95% CI, 1.16-2.26), were more likely to report RCI. Presentation of research at a conference (OR, 1.57; 95% CI, 1.17-2.11) and manuscript authorship (OR, 1.36; 95% CI, 1.01-1.83) prior to medical school were also positively associated with RCI among URiM students. Once matriculated, URiM students who conducted research (OR, 1.57; 95% CI, 1.17-2.12) or authored a manuscript during year 1 (OR, 1.84; 95% CI, 1.04-3.25) were more likely to report RCI. URiM students who identified a physician-scientist role model were also more likely to report RCI (OR, 1.71; 95% CI, 1.25-2.34).

Among the 3 factors found to be associated with RCI in year 1 among non-URiM participants (Figure 3C), one (manuscript authorship prior to medical school) overlapped with factors identified among URiM students. Having a research mentor in year 1 (OR, 1.76; 95% CI, 1.34-2.31) and receiving education about work-life balance of the physician-scientist (OR, 1.63; 95% CI, 1.24-2.15) were associated with RCI among non-URiM students only.

## Discussion

The LEAP study enrolled 1136 first-year students from 134 MD-granting programs across the US to investigate the evolution of RCI during medical school. In year 1, more than one-quarter of first-year medical students reported RCI. The most salient factors associated with RCI among first-year medical students were research experiences both before and after matriculation. We also found differences in factors associated with RCI between URiM and non-URiM students in that some prematriculation research experiences were uniquely associated with RCI among URiM students, whereas identification of a research mentor in year 1 and receipt of information about work-life balance were uniquely associated with RCI among non-URiM students.

We found that most factors associated with RCI in year 1 of medical school were related to research experiences and products (eg, conference presentations and manuscripts) prior to and during medical school. These findings align with social cognitive career theory,^15^ which posits that individuals who engage in activities they feel competent in (ie, research self-efficacy) and expect positive outcomes from (eg, outcome expectations) are more likely to continue participation in the activity. Exposure to research mentors or physician-scientist role models may provide exemplars of successful bridging of clinical care and scientific research for aspiring physician-scientists, which could similarly enhance outcomes expectations. Although we do not yet know how RCI in year 1 will impact RCI at the time of graduation (and beyond), it is promising that some of the factors identified are modifiable, either through tailoring admissions processes to target applicants with research-related experiences or through innovations to research education, engagement, and mentorship during medical school.

We found several differences in factors associated with RCI between URiM and non-URiM students. Intensive pre–medical school research experiences (eg, research-focused gap year and summer research) were associated with RCI among URiM students only. One possible explanation is that non-URiM students may pursue intensive pre–medical school research opportunities for reasons other than interest in a research career (eg, to strengthen competitiveness of medical school applications). It was interesting that research experiences during year 1 were associated with RCI among URiM students, but not among non-URiM students, suggesting that research experiences during medical school may have a greater impact on RCI among URiM students. Finally, our finding that receipt of education about work-life balance of the physician-scientist was associated with RCI among non-URiM students, but not URiM students, suggests that these 2 student groups may use different values in making career decisions. These early findings highlight modifiable levers for intervention.

It is well established that diversity improves the impact and innovation of biomedical research.^42,43,44^ However, a political shift is under way that threatens progress toward diversifying the physician-scientist workforce and will likely affect medical students’ research training. This shift is evident in the recent cancellation of diversity supplement funding and the elimination of NIH training-grant requirements to document efforts to recruit underrepresented trainees. Proposed cuts to research funding overall, along with increased signals of economic unrest, may further endanger the physician-scientist pipeline and disproportionately impact opportunities for aspiring URiM physician-scientists.^45^ In this context, it is imperative that we develop a deep understanding of how RCI evolves during medical school and identify modifiable factors that can be targeted to increase recruitment and retention of URiM and non-URiM trainees to medical research—an imperative under way in LEAP.

### Limitations

This study has some limitations to consider. First, because a focus of this study is URiM physician-scientist development, we recruited a relatively modest cohort of non-URiM students (253 students), which may limit the representativeness of our study findings. Second, although this study recruited students from the vast majority of US medical schools (134 schools), small samples from each school limit our ability to investigate school-level outcomes. Third, the study excluded osteopathic medical students, limiting the generalizability of its findings to this growing student population. Fourth, because of relatively modest sample size, we could not perform split-sample validation of our findings, potentially biasing our *P* values downward. Accordingly, all *P* values should be interpreted as conditional upon variable selection.

## Conclusions

In this cross-sectional analysis of baseline data from the LEAP cohort study, we characterized RCI during the first year of medical school, as well as differences and similarities in factors associated with RCI between URiM and non-URiM medical students early in their medical school careers. Continued follow-up of the LEAP cohort and future analyses will develop trajectories of RCI during medical school and identify characteristics and experiences to explain how and why medical students develop, extinguish, or sustain interest in a research career from matriculation through graduation, informing interventions to build a robust physician-scientist pipeline that reflects the population it serves.

## Notes

Supplement 1. eTable 1. LEAP Assessment Schedule eTable 2. LEAP Baseline Survey Domains and Measure eTable 3. Characteristics of 1st-Year Medical Student Enrollees in LEAP eTable 4. Characteristics of 1st-Year URiM Medical Students with and without RCI eTable 5. Characteristics of 1st-Year non-URiM Medical Students with and without RCI eReferences

Supplement 2. Data Sharing Statement
