# Supplementary material for: Longitudinal Evaluation of Research Career Intentions Among US Medical Students
Source: JAMA Netw Open. 2026 May 12;9(5):e2611430. doi: 10.1001/jamanetworkopen.2026.11430 (PMC13169399; doi:10.1001/jamanetworkopen.2026.11430)
Supplement: Supplement 1. — eTable 1. LEAP Assessment Schedule eTable 2. LEAP Baseline Survey Domains and Measure eTable 3. Characteristics of 1st-Year Medical Student Enrollees in LEAP eTable 4. Characteristics of 1st-Year URiM Medical Students with and without RCI eTable 5. Characteristics of 1st-Year non-URiM Medical Students with and without RCI eReferences [file jamanetwopen-e2611430-s001.pdf]

Supplemental Online Content

Hajduk AM, O'Connell M, Aviles A, et al. Longitudinal evaluation of research career intentions among US medical students. *JAMA Netw Open*. 2026;9(5):e2611430. doi:10.1001/jamanetworkopen.2026.11430

eTable 1. LEAP Assessment Schedule

eTable 2. LEAP Baseline Survey Domains and Measure

eTable 3. Characteristics of 1st-Year Medical Student Enrollees in LEAP

eTable 4. Characteristics of 1st-Year URiM Medical Students with and without RCI

eTable 5. Characteristics of 1st-Year non-URiM Medical Students with and without RCI

eReferences

This supplemental material has been provided by the authors to give readers additional information about their work.

© 2026 Hajduk AM et al. *JAMA Network Open*.

eTable 1. LEAP Assessment Schedule

LEAP Domains, Measures & Timing of Assessments	Year 1	Year 2	Year 3	Year 4	Year 5
**Race[1], Ethnicity[1], URiM Status[2]**	x				
**Sociodemographic Characteristics**					
Age, sex, gender identity, sexual orientation, country & language of origin, childhood socioeconomic disadvantage[3]	x				
Marital status, disability, employment, student debt, financial concerns[4], housing security[5], food security[5]	x	x	x	x	x
**Research Career Intentions[6–8]**	x	x	x	x	x
**Education Prior to Medical School**	x				
Degrees earned<br>MCAT score<br>Satisfaction with premedical school education and training					
**Pre-Medical School Research Experiences**	x				
Research opportunities, types of research conducted, research products, mentorship, and employment in research					
**Medical School Research Experiences**	x	x	x	x	x
Types of research, time allowed, research products, research didactics, academic credit, financial support and motivations for conducting research, satisfaction with research experiences, research skills, experiences finding research mentors, mentor competency (Mentor Competency Assessment)[9], mentor accessibility (Medical Student Scholar-Ideal Mentor Scale)[10,11]					
**Science Identity and Belonging in Research (Modified Scientific Identity Scale ref)** [12,13]	x	x	x	x	x
**Medical School Environment**	x	x	x	x	x

© 2026 Hajduk AM et al. *JAMA Network Open*.

	Medical School Learning Environment Survey[14]<br>Student Sense of School Belonging Scale[5]					
**Diversity, Equity and Inclusion**		x	x	x	x	x
	Medical School Learning Environment Survey[14]<br>Diversity Engagement Survey[15]<br>Race Climate Survey[16]<br>Everyday Discrimination Scale[17]<br>Mistreatment[18]<br>URiM representation and student interaction with URiM medical school leadership<br>Institutional support for URiM students and faculty					
**Other Experiences During Medical School**		x	x	x	x	x
	Academic Standing<br>Competing Life Demands<br>Financial pressure related to career choice<br>Education about the physician-scientist career option<br>Research Scientist role model					
**Psychosocial Variables**		x	x	x	x	x
	Anxiety and Depression, Patient Health Questionnaire-4[19]<br>Grit, Short Grit Scale[20]<br>Burn-out, Maslach Burnout Inventory (2-item)[21]<br>Social Support, Quality of Life[4]<br>Imposter Syndrome, Short Clance Impostor Phenomenon Scale[22]					
**Research Self-efficacy Scale[23]**		x	x	x	x	x
**Outcome Expectations[24]**		x	x	x	x	x
**Qualitative Interviews**		x	x	x	x	x

© 2026 Hajduk AM et al. *JAMA Network Open*.

eTable 2. LEAP Baseline Survey Domains and Measures

Domain	Variables	Categories
**Race & Ethnicity**[1]		
	Hispanic, Latino, Spanish origin	Yes, No
	American Indian or Alaskan Native	Yes, No
	Asian	Yes, No
	Black or African American	Yes, No
	Middle Eastern or North African	Yes, No
	Native Hawaiian or other Pacific Islander	Yes, No
	White	Yes, No
	Other race or ethnicity	Yes, No
**URiM Status**[2]		
	**URiM:** identifying as Black/African American, Hispanic/Latinx, American Indian/Alaska Native, Native Hawaiian/other Pacific Islander multiracial, including one of the above	Yes, No
	**Non-URiM:** identifying as White, Asian, Middle Eastern/North African, Multiracial, including one of the above	Yes, No
**Sociodemographic Characteristics**		
	Age	≤22, 23, 24, 25, 26+
	Sex assigned at birth	Male, Female, prefer not to answer
	Gender identity	Gender diverse, not gender diverse
	Sexual orientation	Heterosexual, Gay/Bisexual/Queer, other
	Country of birth	U.S.-born, non-U.S.-born
	Languages spoken in childhood home	English, Spanish, Other
	Paid employment	Yes, No
	Tuition: family contribution	Yes, No
	Tuition: scholarships	Yes, No

© 2026 Hajduk AM et al. *JAMA Network Open*.

	Loans cover living expenses	Yes, No
	Student debt (current)	None, 1-50k, >50k-100k, >100k
	Socioeconomic disadvantage[2]	Yes, No
	Disability[25]	Yes, No
	Financial insecurity[4]	Yes, No
	Housing insecurity[5]	Yes, No
	Food insecurity[5]	Yes, No
**Research Career Intentions (**AAMC MSQ**)**[1]		
	Activities planned after completing training	Patient Care/ Research/ Teaching/ Administration/ Military Service/ Public Health/ Other
	Level of intended research involvement	Involved in a limited way/significantly, or exclusively involved
**Pre-Medical School Education**		
	STEM major	Yes, No
	MCAT score	Quartiles
	Satisfaction with premedical school education and training	Moderately-very satisfied/Not satisfied
**Pre-Medical School Research Experiences**		
	Participated in a research pathway program	Yes, No
	Held a Paid Research Internship	Yes, No
	Held an Unpaid Research Internship	Yes, No
	Held a Summer Research Position	Yes, No
	Received Course Credit for Research	Yes, No
	1+ Research Gap Year	Yes, No
	Partial Research Gap Year	Yes, No
	Authored a Manuscript	Yes, No
	Oral/Poster Presentation	Yes, No
	Had a research mentor	Yes, No
**Medical School Research Experiences**		
	Authored a Manuscript	Yes, No

© 2026 Hajduk AM et al. *JAMA Network Open*.

Oral/Poster Presentation	Yes, No
Conducted mentored research	Yes, No
Research coursework required	Yes, No
Required to conduct research	Yes, No
Research coursework required	Yes, No/unsure
Required to conduct research	Yes, No/unsure
Availability of <u>financial support</u> for student research	
Summer Research Stipend	Yes, No/unsure
Full-Year Research Stipend	Yes, No/unsure
Financial Support for Research Experiences	Yes, No/unsure
Funds to Travel to a Research Meeting	Yes, No/unsure
Full-time Research Opportunities	Yes, No/unsure
<u>Primary</u> reason for conducting research	Percent endorsing
Develop Technical/Transferable Skills	
Satisfy Intellectual Curiosity	
Satisfy Curricular/Graduation Requirements	
Increase Competitiveness for Residency	
Contribute to Science Advancements	
Enhance Health Equity	
Provide Time to Pursue Other Opportunities	
Delay Other Clinical/Academic Duties	
Support Career Goal of Being a Researcher	
Other	
Research skill development[9]	
Research experience taught me to…	
Ask Questions	Yes, No
Analyze Data	Yes, No
Use New Techniques	Yes, No

© 2026 Hajduk AM et al. *JAMA Network Open*.

	Develop Methods	Yes, No
	Critically Review Literature	Yes, No
	Write a Manuscript	Yes, No
	Incorporate Guidelines	Yes, No
	None of the Above	Yes, No
	Mentorship Quality	Score on six competency subscales
	Mentor Competency Assessment[9]	Effective communication<br>Aligning expectations<br>Assessing understanding<br>Addressing diversity<br>Fostering independence<br>Promoting professional development
	Mentor Accessibility	
	Medical Student Scholar-Ideal Mentor Scale[11]	
	Satisfaction with primary research mentor's response to email	
	Satisfaction with the frequency with which primary research mentor meets with you	
**Science Identity and Belonging in Research**		
	Modified Scientific Identity Scale[12]	Composite score, average of items
**Medical School Learning Environment**		
	Medical School Learning Environment Survey[14]	Average score, ordinal
	Student Sense of School Belonging Scale[5]	Average score<br>Low sense of belonging (≤7.3)<br>Some sense of belonging (7.4-9.6)<br>High sense of belonging (≥9.7)
**Diversity, Equity and Inclusion**		
	Institutional Diversity & Inclusion	
	Diversity Engagement Survey[15]	Global score, ordinal
	Race Climate Survey[16]	Subscale score, ordinal
	Everyday Discrimination Scale[17]	Composite ordinal scale

© 2026 Hajduk AM et al. *JAMA Network Open*.

	Mistreatment[18]	Any report of mistreatment in each domain
	Sexual Orientation Discrimination	
	Race Discrimination	
	Sex Discrimination	
	URiM representation in education, research or administrative leadership	Yes, No
	Institutional support for URiM students and faculty	5 pt: Not at all supportive-supportive
**Experiences During Medical School (other)**		
	Competing Life Demands (impact on time for school & research)	
	Activism	Yes, No
	Committee work	Yes, No
	DEI work	Yes, No
	Family	Yes, No
	Employment	Yes, No
	Volunteer work	Yes, No
	Importance of compensation in career choice	5 pt: Not at all important-very important
	Received education about factors involved in the physician-scientist pathway:	
	Satisfaction of intellectual curiosity	Yes, No
	Potential of work to influence patient care	Yes, No
	Employment opportunities	Yes, No
	Compensation	Yes, No
	Work-life balance	Yes, No
	Autonomy	Yes, No
	Funding opportunities	Yes, No
	Prolonged training/years of education	Yes, No

© 2026 Hajduk AM et al. *JAMA Network Open*.

	Medical school presents a research scientist career path	5pt: Very negatively-very
	Has a physician-scientist role model	Yes, No
**Psychosocial Variables**		
	Patient Health Questionnaire-4[19]	
	Anxiety symptoms-2 items	Yes (score >2), No
	Depressive symptoms-2 items	Yes (score >2), No
	Grit	
	Short Grit Scale[20]	Composite ordinal score
	Burnout (burned out)	
	2-item Maslach Burnout Inventory[21]	Yes (endorsement of either item), No
	Social Support	
	School peers considered friends;<br>URiM individuals in the research network	Average number
	Quality of Life (AAMC Y2Q)[4]	
	Rate overall wellbeing past week 0-10 (as bad as it can be-as good as it can be)	Average score
	Imposter Syndrome	
	Short Clance Impostor Phenomenon Scale[22]	Average score, continuous
**Potential Mediators of Research Career Intentions**		
	Research self-efficacy[23]	Summary score
	Outcome expectations for research career[24]	Average score

© 2026 Hajduk AM et al. *JAMA Network Open*.

eTable 3. Characteristics of 1st-Year Medical Student Enrollees in LEAP

Characteristics	N(%) or mean (SD)			SMD
	All (n=1136)	URiM (n=883)	Non-URiM (n=253)	
**Sex**				0.059
Female	790 (69.5)	617 (69.9)	173 (68.4)	
Assigned male sex at birth	345 (30.4)	265 (30.0)	80 (31.6)	
Prefer not to answer	1 (0.1)	1 (0.1)	0 (0.0)	
**Paid Employment**				0.114
No	954 (84.0)	750 (84.9)	204 (80.6)	
Yes	178 (15.7)	130 (14.7)	48 (19.0)	
**From a Disadvantaged Background**	515 (45.3)	451 (51.1)	64 (25.3)	0.550
**Race/Ethnicity (non-exclusive)***				
American Indian	29 (2.6)	29 (3.3)	0 (0.0)	0.261
Hawaiian Pacific	19 (1.7)	19 (2.2)	0 (0.0)	0.210
Middle East	43 (3.8)	22 (2.5)	21 (8.3)	0.259
Black	514 (45.2)	514 (58.2)	0 (0.0)	1.669
Hispanic	359 (31.6)	359 (40.7)	0 (0.0)	1.171
White	292 (25.7)	156 (17.7)	136 (53.8)	0.813
Asian	162 (14.3)	47 (5.3)	115 (45.5)	1.039
Other	20 (1.80)	16 (1.80)	4 (1.60)	0.018
**Born outside the US**	233 (20.5)	198 (22.4)	35 (13.8)	0.237
**Age ≤ 25 years old**	830 (73.1)	621 (70.3)	209 (82.6)	0.377
**Not Gender Diverse**	1105 (97.3)	859 (97.3)	246 (97.2)	0.017
**Heterosexual Orientation**	925 (81.4)	728 (82.4)	197 (77.9)	0.127
**Native Language**				0.896
English	773 (68.0)	594 (67.3)	179 (70.8)	
Spanish	196 (17.3)	195 (22.1)	1 (0.4)	
Other	139 (12.2)	67 (7.6))	72 (28.5)	
**Financial Insecurity**	526 (46.3)	431 (48.8)	95 (37.5)	0.224
**Food Insecurity**	222 (19.5)	198 (22.4)	24 (9.5)	0.359

© 2026 Hajduk AM et al. *JAMA Network Open*.

**Housing Insecurity**	14 (1.2)	11 (1.2)	3 (1.2)	0.005
**Tuition is covered by:**				
Family tuition support	258 (22.7)	145 (16.4)	113 (44.7)	0.650
Scholarships	658 (57.9)	529 (59.9)	129 (51.0)	0.178
**Educational Debt**				0.491
No debt	193 (17.0)	114 (12.9)	79 (31.2)	
1-50k	286 (25.2)	234 (26.5)	52 (20.6)	
>50k-100k	337 (29.7)	265 (30.0)	72 (28.5)	
>100k	310 (27.3)	264 (29.9)	46 (18.2)	
**Living expenses are covered by other loans**	249 (21.9)	206 (23.3)	43 (17.0)	0.158
**Has a Disability**	83 (7.3)	68 (7.7)	15 (5.9)	0.071
**Prior to medical school, the student:**				
Gave an oral/poster presentation	470 (41.4)	363 (41.1)	107 (42.3)	0.024
Authored a manuscript	412 (36.3)	304 (34.4)	108 (42.7)	0.170
Obtained MCAT score between:				1.060
481-504	319 (28.1)	304 (34.4)	15 (5.9)	
505-509	264 (23.2)	225 (25.5)	39 (15.4)	
510-514	234 (20.6)	169 (19.1)	65 (25.7)	
515-527	258 (22.7)	133 (15.1)	125 (49.4)	
Participated in a research pathway program	123 (10.8)	107 (12.1)	16 (6.3)	0.201
Held a paid research internship	400 (35.2)	288 (32.6)	112 (44.3)	0.241
Held an unpaid research internship	578 (50.9)	422 (47.8)	156 (61.7)	0.281
Held a summer research position	456 (40.1)	348 (39.4)	108 (42.7)	0.067
Received course credit for research	513 (45.2)	369 (41.8)	144 (56.9)	0.306
Took 1+ research gap year	226 (19.9)	175 (19.8)	51 (20.2)	0.008
Took a partial research gap year	143 (12.6)	116 (13.1)	27 (10.7)	0.076
Felt satisfied with pre-med education	935 (82.3)	709 (80.3)	226 (89.3)	0.251
Obtained a background in STEM	956 (84.2)	735 (83.2)	221 (87.4)	0.096
**Student Reported Feeling:**				
Burnout	396 (34.9)	319 (36.1)	77 (30.4)	0.121
Grit, mean (SD)	3.74 (0.57)	3.75 (0.58)	3.69 (0.53)	0.097

© 2026 Hajduk AM et al. *JAMA Network Open*.

Imposter Syndrome, mean (SD)<br>Anxiety symptoms<br>Depression symptoms<br>Student feels they can rely on peers, mean (SD)<br># of peers whom they consider friends, mean (SD)<br># of friends who are URiM, mean (SD)<br>Overall wellbeing, mean (SD)<br>Financial compensation is important<br>A sense of belonging in science, mean (SD)	2.97 (0.93)<br>428 (37.7)<br>157 (13.8)<br>4.69 (2.90)<br>6.88 (3.02)<br>4.93 (3.37)<br>6.17 (1.95)<br>910 (80.1)<br>3.01 (0.86)	2.98 (0.93)<br>344 (39.0)<br>121 (13.7)<br>4.52 (2.85)<br>6.68 (3.04)<br>5.27 (3.34)<br>6.18 (1.99)<br>719 (81.4)<br>3.03 (0.88)	2.94 (0.91)<br>84 (33.2)<br>36 (14.2)<br>5.31 (2.99)<br>7.59 (2.86)<br>3.71 (3.20)<br>6.15 (1.77)<br>191 (75.5)<br>2.97 (0.77)	0.047<br>0.122<br>0.015<br>0.270<br>0.308<br>0.476<br>0.014<br>0.253<br>0.068
**Research scientist career path is positively presented**	629 (55.4)	494 (55.9)	135 (53.4)	0.189
**Research-related coursework is required in the medical school curriculum**	556 (4.9)	441 (49.9)	115 (45.5)	0.090
**Required to conduct research during medical school**	543 (47.8)	440 (49.8)	103 (40.7)	0.184
**Received information that the physician scientist pathway included:**<br>Satisfaction of intellectual curiosity<br>Potential of work to influence patient care<br>Employment opportunities<br>Compensation<br>Work-life balance<br>Work-work balance<br>Autonomy<br>Funding opportunities<br>Prolonged training/years of education	 <br>561 (49.4)<br>741 (65.2)<br>344 (30.3)<br>252 (22.2)<br>425 (37.4)<br>435 (38.3)<br>343 (30.2)<br>407 (35.8)<br>620 (54.6)	 <br>442 (50.1)<br>583 (66.0)<br>280 (31.7)<br>209 (23.7)<br>338 (38.3)<br>350 (39.6)<br>279 (31.6)<br>327 (37.0)<br>493 (55.8)	 <br>119 (47.0)<br>158 (62.50)<br>64 (25.30)<br>43 (17)<br>87 (34.4)<br>85 (33.6)<br>64 (25.30)<br>80 (31.6)<br>127 (50.2)	 <br>0.064<br>0.082<br>0.146<br>0.169<br>0.085<br>0.130<br>0.138<br>0.112<br>0.116
**During medical school, the student has:**<br>Authored a manuscript<br>Conducted research<br>Presented research findings<br>A physician-scientist role model<br>Participated in formal research coursework	 62 (5.5)<br>396 (34.9)<br>57 (5.0)<br>270 (23.8)<br>64 (5.6)	 50 (5.7)<br>305 (34.5)<br>49 (5.5)<br>212 (24.0)<br>48 (5.4)	 12 (4.7)<br>91 (36.0)<br>8 (3.2)<br>58 (22.9)<br>16 (6.3)	 0.041<br>0.033<br>0.117<br>0.029<br>0.038
**Proposed Mediators of Research Career Intention***				

© 2026 Hajduk AM et al. *JAMA Network Open*.

Research self-efficacy	34.11 (5.93)	34.00 (6.02)	34.47 (5.60)	0.080
Outcomes expectations	3.51 (0.80)	3.56 (0.81)	3.35 (0.74)	0.263
**Medical students are offered:**				
Financial support for student research	648 (57.0)	495 (56.1)	153 (60.5)	0.087
Summer research stipend	577 (50.8)	436 (49.4)	141 (55.7)	0.128
Full-Year research stipend	125 (11.0)	98 (11.1)	27 (10.7)	0.014
Financial support for research expenses	161 (14.2)	119 (13.5)	42 (16.6)	0.087
Funds to travel to a research meeting	313 (27.6)	236 (26.7)	77 (30.4)	0.082
Opportunities for full-time research	464 (40.8)	349 (39.5)	115 (45.5)	0.603
**Primary reason for conducting research:**				0.250
Develop technical/transferable skills	63 (5.5)	50 (5.7)	13 (5.1)	
Satisfy intellectual curiosity	63 (5.5)	45 (5.1)	18 (7.1)	
Satisfy curricular/graduation requirements	70 (6.2)	57 (6.5)	13 (5.1)	
Increase competitiveness for residency	369 (32.5)	284 (32.2)	85 (33.6)	
Contribute to scientific advancements	38 (3.3)	30 (3.4)	8 (3.2)	
Enhance health equity	112 (9.9)	96 (10.9)	16 (6.3)	
Provide time to pursue other opportunities	5 (0.4)	5 (0.6)	0 (0.0)	
Take a break from other clinical/academic duties	4 (0.4)	3 (0.3)	1 (0.4)	
Support career goal of being a researcher	14 (1.2)	12 (1.4)	2 (0.8)	
Other	3 (0.3)	3 (0.3)	0 (0.0)	
**The research scientist career path is presented:**				0.189
Very negatively	4 (0.4)	4 (0.5)	0 (0)	
Somewhat negatively	35 (3.1)	22 (2.5)	13 (5.1)	
Neither	366 (32.2)	276 (31.3)	90 (35.6)	
Somewhat positively	372 (32.7)	293 (33.2)	79 (31.2)	
Very positively	257 (22.6)	201 (22.8)	56 (22.1)	
**Medical school research experience taught student to:**				
Ask questions	260 (22.9)	204 (23.1)	56 (22.1)	0.084
Analyze data	274 (24.1)	205 (23.2)	69 (27.3)	0.109
Use new techniques	193 (17.0)	155 (17.6)	38 (15.0)	0.120
Develop methods	184 (16.2)	140 (15.9)	44 (17.4)	0.044

© 2026 Hajduk AM et al. *JAMA Network Open*.

Critically review literature	264 (23.2)	207 (23.4)	57 (22.5)	0.083
Write a manuscript	159 (14.0)	128 (14.5)	31 (12.3)	0.109
Incorporate guidelines	143 (12.6)	109 (12.3)	34 (13.4)	0.041
None of the above	13 (1.1)	13 (1.5)	0 (0)	0.183
**The student has a research mentor**	526 (46.4)	421 (47.8)	105 (41.5)	0.127
**Student's research mentor is skilled in:**				
Employing strategies to enhance research understanding				0.167
Yes	430 (37.9)	350 (39.6)	80 (31.6)	
No	87 (7.7)	67 (7.6)	20 (7.9)	
N/A	602 (53.0)	454 (51.4)	148 (58.5)	
Maintaining effective communication				0.143
Yes	451 (39.7)	362 (41.0)	89 (35.2)	
No	68 (6.0)	55 (6.2)	13 (5.1)	
N/A	602 (53.0)	454 (51.4)	148 (58.5)	
Addressing diversity				0.162
Yes	401 (35.3)	326 (36.9)	75 (29.6)	
No	120 (10.6)	95 (10.8)	25 (9.9)	
N/A	602 (53.0)	454 (51.4)	148 (58.5)	
Aligning expectations				0.139
Yes	451 (39.7)	362 (41.0)	89 (35.2)	
No	70 (6.2)	56 (6.3)	14 (5.5)	
N/A	602 (53.0)	454 (51.4)	148 (58.5)	
Fostering independence				0.145
Yes	438 (38.6)	353 (40.0)	85 (33.6)	
No	81 (7.1)	64 (7.2)	17 (6.7)	
N/A	602 (53.0)	454 (51.4)	148 (58.5)	
Promoting professional development				0.167
Yes	414 (36.4)	331 (37.5)	83 (32.8)	
No	104 (9.2)	87 (9.9)	17 (6.7)	
N/A	602 (53.0)	454 (51.4)	148 (58.5)	
Answering emails				0.142

© 2026 Hajduk AM et al. *JAMA Network Open*.

Very Dissatisfied	4 (0.4)	3 (0.3)	1 (0.4)	
Dissatisfied	22 (1.9)	18 (2.0)	4 (1.6)	
Neutral	61 (5.4)	48 (5.4)	13 (5.1)	
Satisfied	187 (16.5)	151 (17.1)	36 (14.2)	
Very Satisfied	251 (22.1)	202 (22.9)	49 (19.4)	
N/A	608 (53.5)	460 (52.1)	148 (58.5)	
Meeting regularly with mentee				0.213
Very Dissatisfied	10 (0.9)	9 (1.0)	1 (0.4)	
Dissatisfied	19 (1.7)	18 (2.0)	1 (0.4)	
Neutral	103 (9.1)	84 (9.5)	19 (7.5)	
Satisfied	183 (16.1)	142 (16.1)	41 (16.2)	
Very Satisfied	210 (18.5)	169 (19.1)	41 (16.2)	
N/A	608 (53.5)	460 (52.1)	148 (58.5)	
MCA score				0.167
≤4	87 (7.7)	67 (7.6)	20 (7.9)	
>4	430 (37.9)	350 (39.6)	80 (31.6)	
N/A (no mentor)	602 (53.0)	454 (51.4)	148 (58.5)	
**School Climate:**				
MSLES	3.75 (0.45)	3.73 (0.47)	3.82 (0.39)	0.196
DES Score	4.04 (0.60)	4.02 (0.61)	4.10 (0.54)	0.138
Everyday Discrimination Scale	1.90 (0.85)	1.99 (0.88)	1.59 (0.66)	-0.518
Experience of Orientation-based Discrimination	54 (4.8)	44 (5.0)	10 (4.0)	0.050
Experience of Race-based Discrimination	191 (16.8)	164 (18.6)	27 (10.7)	0.225
Experience of Sex-based Discrimination	128 (11.3)	103 (11.7)	25 (9.9)	0.058
Mistreatment	261 (23.0)	217 (24.6)	44 (17.4)	0.177
Race Climate	5.59 (1.09)	5.52 (1.12)	5.81 (0.95)	0.282
Institution is URiM supportive	932 (82.0)	723 (81.9)	209 (82.6)	0.264
Institution has URiM Leadership	993 (87.4)	775 (87.8)	218 (86.2)	0.054
Student reports sense of belonging as:				0.133
Low	46 (4.0)	40 (4.5)	6 (2.4)	
Some	92 (8.1)	74 (8.4)	18 (7.1)	

© 2026 Hajduk AM et al. *JAMA Network Open*.

High	984 (86.6)	757 (85.7)	227 (89.7)	
**The student has limited time to engage in academic pursuits due to:**	104 (9.2)	85 (9.6)	19 (7.5)	0.094
Outside Activism	122 (10.7)	100 (11.3)	22 (8.7)	0.113
Outside Committee	152 (13.4)	136 (15.4)	16 (6.3)	0.320
Outside DEI work	195 (17.2)	160 (18.1)	35 (13.8)	0.138
Outside Family	90 (7.9)	69 (7.8)	21 (8.3)	0.014
Outside Employment	241 (21.2)	188 (21.3)	53 (20.9)	0.029
Outside Volunteer	241 (21.2)	188 (21.3)	53 (20.9)	0.029

*not included as a candidate variable in regression models; include for descriptive purposes only

© 2026 Hajduk AM et al. *JAMA Network Open*.

eTable 4. Characteristics of 1st-Year URiM Medical Students with and without RCI

Characteristics	N(%) or mean (SD)		SMD
	**No RCI N=637**	**RCI N=246**	
**Assigned Female Sex at Birth**	451 (70.8)	166 (67.5)	0.093
**Paid Employment**			0.019
No	540 (84.8)	210 (85.4)	
Yes	95 (14.9)	35 (14.2)	
**From a Disadvantaged Background**	319 (50.1)	132 (53.7)	0.072
**Race/Ethnicity (non-exclusive)**			
American Indian	23 (3.6)	6 (2.4)	0.068
Hawaiian Pacific	12 (1.9)	7 (2.8)	0.063
Middle East	15 (2.4)	7 (2.8)	0.031
Black	386 (60.6)	128 (52.0)	0.173
Hispanic	240 (37.7)	119 (48.4)	0.217
White	108 (17.0)	48 (19.5)	0.066
Asian	35 (5.5)	12 (4.9)	0.028
Other	8 (1.3)	8 (3.3)	0.135
**Born Outside the US**	137 (21.5)	61 (24.8)	0.091
**Age ≤ 25 Years Old**	460 (72.2)	161 (65.5)	0.194
**Not Gender Diverse**	621 (97.5)	238 (96.7)	0.064
**Heterosexual Orientation**	535 (84.0)	193 (78.5)	0.143
**Native Language**			0.207
English	448 (70.3)	146 (59.3)	
Spanish	130 (20.4)	65 (26.4)	
Other	44 (6.9)	23 (9.3)	
**Financial Insecurity**	30 (47.7)	127 (51.6)	0.072
**Food Insecurity**	130 (20.4)	68 (27.6)	0.168
**Housing Insecurity**	7 (1.1)	4 (1.6)	0.045
**Tuition is covered by:**			

© 2026 Hajduk AM et al. *JAMA Network Open*.

Family tuition support	106 (16.6)	39 (15.9)	0.024
Scholarships	388 (60.9)	141 (57.3)	0.074
**Educational Debt**			0.163
No debt	90 (14.1)	24 (9.8)	
1-50k	172 (27.0)	62 (25.2)	
>50k-100k	191 (30.0)	74 (30.1)	
>100k	181 (28.4)	83 (33.7)	
**Living expenses are covered by other loans**	147 (23.1)	59 (24.0)	0.021
**Has a Disability**	46 (7.2)	22 (8.9)	0.063
**Prior to medical school:**			
Gave an oral/poster presentation	226 (35.5)	137 (55.7)	0.414
Authored a manuscript	190 (29.8)	114 (46.3)	0.345
Obtained MCAT score between:			0.173
481-504	216 (33.9)	88 (35.8)	
505-509	169 (26.5)	56 (22.8)	
510-514	129 (20.3)	40 (16.3)	
515-527	88 (13.8)	45 (18.3)	
Participated in a research pathway program	66 (10.4)	41 (16.7)	0.185
Held a paid research internship	184 (28.9)	104 (42.3)	0.282
Held an unpaid research internship	299 (46.9)	123 (50.0)	0.061
Held a summer research position	223 (35.0)	125 (50.8)	0.323
Received course credit for research	252 (39.6)	117 (47.6)	0.162
Took 1+ research gap year	100 (15.7)	75 (30.5)	0.356
Took a partial research gap year	73 (11.5)	43 (17.5)	0.172
Felt satisfied with pre-med education	506 (79.4)	203 (82.5)	0.088
Obtained a background in STEM	528 (82.9)	207 (84.1)	0.019
**Student Reported Feeling:**			
Burnout	221 (34.7)	98 (39.8)	0.107
Grit	3.71 (0.59)	3.84 (0.55)	-0.231
Imposter Syndrome	3.01 (0.93)	2.90 (0.93)	0.120
Anxiety symptoms	239 (37.5)	105 (42.7)	0.103

© 2026 Hajduk AM et al. *JAMA Network Open*.

Depression symptoms	83 (13.0)	38 (15.4)	0.070
Student feels they can rely on peers	4.46 (2.85)	4.66 (2.87)	-0.069
The student has peers whom they consider friends	6.65 (3.02)	6.78 (3.10)	-0.043
The student has friends who are URiM	5.22 (3.34)	5.41 (3.35)	-0.059
Overall wellbeing	6.22 (1.98)	6.09 (2.04)	0.065
Financial compensation is important	515 (80.9)	204 (82.9)	0.085
A sense of belonging in science	2.82 (0.83)	3.55 (0.78)	-0.898
**Research scientist career path is positively presented**	352 (55.3)	142 (57.7)	0.117
**Research-related coursework is required in the medical school curriculum**	302 (47.4)	139 (56.5)	0.183
**Required to conduct research during medical school**	319 (50.1)	121 (49.2)	0.018
**Received information that the physician scientist pathway included:**			
Satisfaction of intellectual curiosity	308 (48.4)	134 (54.5)	0.130
Potential of work to influence patient care	406 (63.7)	177 (72.0)	0.186
Employment opportunities	201 (31.6)	79 (32.1)	0.018
Compensation	151 (23.7)	58 (23.6)	0.000
Work-life balance	224 (35.2)	114 (46.3)	0.234
Work-work balance	234 (36.7)	116 (47.2)	0.223
Autonomy	187 (29.4)	92 (37.4)	0.177
Funding opportunities	233 (36.6)	94 (38.2)	0.039
Prolonged training/years of education	356 (55.9)	137 (55.7)	0.005
**During Medical School, the student has:**			
Authored a manuscript	23 (3.6)	27 (11.0)	0.286
Conducted research	185 (29.0)	120 (48.8)	0.413
Presented research findings	28 (4.4)	21 (8.5)	0.169
A physician-scientist role model	124 (19.5)	88 (35.8)	0.371
Participated in formal research coursework	33 (5.2)	15 (6.1)	0.040
**Proposed Mediators of Research Career Intention**			
Research Self-efficacy	33.06(6.00)	36.40(5.38)	0.586
Outcomes Expectations	3.42 (0.81)	3.90 (0.69)	0.639
**Medical Students are Offered:**			
Financial support for student research	352 (55.3)	143 (58.1)	0.054

© 2026 Hajduk AM et al. *JAMA Network Open*.

Summer research stipend	313 (49.1)	123 (50.0)	0.017
Full-Year research stipend	67 (10.5)	31 (12.6)	0.065
Financial support for research expenses	86 (13.5)	33 (13.4)	0.003
Funds to travel to a research meeting	158 (24.8)	78 (31.7)	0.154
Opportunities for full-time research	244 (38.3)	105 (42.7)	0.145
**Primary Reason for Conducting Research:**			0.489
Develop technical/transferable skills	35 (5.5)	15 (6.1)	
Satisfy intellectual curiosity	20 (3.1)	25 (10.2)	
Satisfy curricular/graduation requirements	48 (7.5)	9 (3.7)	
Increase competitiveness for residency	206 (32.3)	78 (31.7)	
Contribute to scientific advancements	17 (2.7)	13 (5.3)	
Enhance health equity	65 (10.2)	31 (12.6)	
Provide time to pursue other opportunities	3 (0.5)	2 (0.8)	
Take a break from other clinical/academic duties	3 (0.5)	0 (0.0)	
Support career goal of being a researcher	3 (0.5)	9 (3.7)	
Other	2 (0.3)	1 (0.4)	
**The research scientist career path is presented:**			0.117
Very negatively	3 (0.5)	1 (0.4)	
Somewhat negatively	16 (2.5)	6 (2.4)	
Neither	199 (31.2)	77 (31.3)	
Somewhat positively	216 (33.9)	77 (31.3)	
Very positively	136 (21.4)	65 (26.4)	
**Medical School Research Experience Taught How To:**			
Ask questions	117 (18.4)	87 (35.4)	0.431
Analyze data	121 (19.0)	84 (34.1)	0.418
Use new techniques	93 (14.8)	61 (24.8)	0.413
Develop methods	85 (13.3)	55 (22.4)	0.413
Critically review literature	121 (19.0)	86 (35.0)	0.422
Write a manuscript	67 (10.5)	61 (24.8)	0.451
Incorporate guidelines	62 (9.7)	47 (19.1)	0.420
None of the above	10 (1.6)	3 (1.2)	0.424

© 2026 Hajduk AM et al. *JAMA Network Open*.

**The student has a research mentor**	280 (44.0)	143 (58.1)	0.286
**Student's research mentor is skilled in:**			
Employing strategies to enhance research understanding			0.366
Yes	223 (35.0)	127 (51.6)	
No	55 (8.6)	12 (4.9)	
N/A	353 (55.4)	101 (41.1)	
Maintaining effective communication			0.297
Yes	237 (37.2)	125 (50.8)	
No	40 (6.3)	15 (6.1)	
N/A	353 (55.4)	101 (41.1)	
Addressing diversity			0.318
Yes	209 (32.8)	117 (47.6)	
No	70 (11.0)	25 (10.2)	
N/A	353 (55.4)	101 (41.1)	
Aligning expectations			0.368
Yes	230 (36.1)	132 (53.7)	
No	46 (7.2)	10 (4.1)	
N/A	353 (55.4)	101 (41.1)	
Fostering independence			0.301
Yes	230 (36.1)	123 (50.0)	
No	46 (7.2)	18 (7.3)	
N/A	353 (55.4)	101 (41.1)	
Promoting professional development			0.314
Yes	213 (33.4)	118 (48.0)	
No	64 (10.0)	23 (9.3)	
N/A	353 (55.4)	101 (41.1)	
Answering emails			0.333
Very Dissatisfied	3 (0.5)	0 (0.0)	
Dissatisfied	11 (1.7)	7 (2.8)	
Neutral	36 (5.7)	12 (4.9)	
Satisfied	101 (15.9)	50 (20.3)	

© 2026 Hajduk AM et al. *JAMA Network Open*.

Very Satisfied	128 (20.1)	74 (30.1)	
N/A	357 (56.0)	103 (41.9)	
Meeting regularly with mentee			0.338
Very Dissatisfied	6 (0.9)	3 (1.2)	
Dissatisfied	10 (1.6)	8 (3.3)	
Neutral	64 (10.0)	20 (8.1)	
Satisfied	90 (14.1)	52 (21.1)	
Very Satisfied	109 (17.1)	60 (24.4)	
N/A	357 (56.0)	103 (41.9)	
MCA score			0.319
N/A (no mentor)	231 (36.3)	124 (50.4)	
≤4	38 (6.0)	10 (4.1)	
>4	353 (55.4)	101 (41.1)	
**School Climate:**			
MSLES	3.73 (0.46)	3.73 (0.48)	-0.000
DES Score	4.01 (0.60)	4.05 (0.63)	-0.066
Everyday Discrimination Scale	1.98 (0.85)	2.03 (0.97)	-0.063
Experience of Orientation-based Discrimination	29 (4.6)	15 (6.1)	0.069
Experience of Race-based Discrimination	108 (17.0)	56 (22.8)	0.146
Experience of Sex-based Discrimination	62 (9.7)	41 (16.7)	0.205
Mistreatment	145 (22.8)	72 (29.3)	0.149
Race Climate	5.55 (1.10)	5.46 (1.16)	0.077
Institution is URiM supportive	519 (81.5)	204 (82.9)	0.116
Institution has URiM Leadership	558 (87.6)	217 (88.2)	0.010
Student reports sense of belonging as:			0.145
Low	29 (4.6)	11 (4.5)	
Some	60 (9.4)	14 (5.7)	
High	538 (84.5)	219 (89.0)	
**The student has limited time to engage in academic pursuits due to:**			
Outside activism	58 (9.1)	27 (11.0)	0.070
Outside committee	71 (11.1)	29 (11.8)	0.021

© 2026 Hajduk AM et al. *JAMA Network Open*.

Outside DEI work	95 (14.9)	41 (16.7)	0.048
Outside family	119 (18.7)	41 (16.7)	0.049
Outside employment	47 (7.4)	22 (8.9)	0.062
Outside volunteer	141 (22.1)	47 (19.1)	0.066

© 2026 Hajduk AM et al. *JAMA Network Open*.

eTable 5. Characteristics of 1st-Year Non-URiM Medical Students with and without RCI

Characteristics	N (%) or mean(SD)		SMD
	**No RCI (n=193)**	**RCI (n=60)**	
**Assigned female sex at birth**	134 (69.4)	39 (65.0)	0.094
**From a Disadvantaged Background**	44 (22.8)	20 (33.3)	0.110
**Race/Ethnicity (non-exclusive)**			
American Indian	0 (0.0)	0 (0.0)	
Hawaiian Pacific	0 (0.0)	0 (0.0)	
Middle East	15 (7.8)	6 (10.0)	0.078
Black	0 (0.0)	0 (0.0)	
Hispanic	0 (0.0)	0 (0.0)	
White	108 (56.0)	28 (46.7)	0.187
Asian	84 (43.5)	31 (51.7)	0.164
Other	4 (2.1)	0 (0.0)	0.206
**Paid Employment**			
No	157 (81.3)	47 (78.3)	0.043
Yes	36 (18.7)	12 (20.0)	
**Has a Disability**	10 (5.2)	5 (8.3)	0.126
**Born Outside the US**	23 (11.9)	12 (20.0)	0. 220
**Age ≤ 25 Years Old**	158 (81.9)	51 (85.1)	0.290
**Not Gender Diverse**	187 (96.9)	59 (98.3)	0.095
**Heterosexual Orientation**	149 (77.2)	48 (80.0)	0.294
**Language**			
English	139 (72.0)	40(66.7)	0.168
Spanish	1 (0.5)	0 (0.0)	
Other	52 (26.9)	20 (33.3)	
**Financial Insecurity**	67 (34.7)	28 (46.7)	0.233
**Food Insecurity**	16 (8.3)	8 (13.3)	0.161
**Housing Insecurity**	2 (1.0)	1 (1.7)	0.055

© 2026 Hajduk AM et al. *JAMA Network Open*.

**Tuition is covered by:**			
Family tuition support	86 (44.6)	27 (45.0)	0.001
Scholarships	101 (52.3)	28 (46.7)	0.125
**Educational Debt**			0.097
No debt	61 (31.6)	18 (30.0)	
1-50k	38 (19.7)	14 (23.3)	
>50k-100k	55 (28.5)	17 (28.3)	
>100k	36 (18.7)	10 (16.7)	
**Living expenses are covered by other loans**	33 (17.1)	10 (16.7)	0.012
**Prior to medical school, the student:**			
Gave an oral/poster presentation	74 (38.3)	33 (55.0)	0.339
Authored a manuscript	73 (37.8)	35 (58.3)	0.419
Obtained an MCAT score between:			0.185
481-504	11 (5.7)	4 (6.7)	
505-509	32 (16.6)	7 (11.7)	
510-514	51 (26.4)	14 (23.3)	
515-527	92 (47.7)	33 (55.0)	
Participated in a research pathway program	11 (5.7)	5 (8.3)	0.103
Held a paid research internship	79 (40.9)	33 (55.0)	0.284
Held an unpaid research internship	112 (58.0)	44 (73.3)	0.327
Held a summer research position	74 (38.3)	34 (56.7)	0.373
Received course credit for research	109 (56.5)	35 (58.3)	0.038
Took 1+ research gap year	32 (16.6)	19 (31.7)	0.358
Took a partial research gap year	19 (9.8)	8(13.3)	0.109
Felt satisfied with pre-med education	168 (87.0)	58 (96.7)	0.357
Obtained a background in STEM	166 (86.0)	55 (91.7)	0.180
**Student Reported Feeling:**			
Burnout	61 (31.6)	16 (26.7)	0.109
Grit	3.66 (0.53)	3.80 (0.52)	-0.256
Imposter syndrome	3.00 (0.91)	2.73 (0.87)	0.297
Anxiety symptoms	67 (34.7)	17 (28.3)	0.138

© 2026 Hajduk AM et al. *JAMA Network Open*.

Depression symptoms	28 (14.5)	8 (13.3)	0.036
Student feels they can rely on peers	5.22 (2.93)	5.57 (3.17)	-0.112
The student has peers whom they consider friends	7.57 (2.83)	7.67 (2.97)	-0.034
The student has friends who are URiM	3.64 (3.15)	3.95 (3.40)	-0.095
Overall wellbeing	6.08 (1.79)	6.40 (1.71)	-0.184
Financial compensation is important	148 (76.7)	43 (71.7)	0.447
A sense of belonging in science	2.80 (0.69)	3.51 (0.78)	-0.969
**The research scientist career path is positively presented**	97 (50.2)	38 (63.3)	0.249
**Research-related coursework is required in the medical school curriculum**	89 (46.1)	26 (43.3)	0.056
**Required to conduct research during medical school**	78 (40.4)	25 (41.7)	0.025
**Received Information that the Physician Scientist Pathway included:**			
Satisfaction of intellectual curiosity	82 (42.5)	37 (61.7)	0.391
Potential of work to influence patient care	112 (58.0)	46 (76.7)	0.399
Employment opportunities	47 (24.4)	17 (28.3)	0.090
Compensation	31 (16.1)	12 (20.0)	0.103
Work-life balance	57 (29.5)	30 (50.0)	0.428
Work-work balance	63 (32.6)	22 (36.7)	0.081
Autonomy	42 (21.8)	22 (36.7)	0.327
Funding opportunities	57 (29.5)	23 (38.3)	0.193
Prolonged training/years of education	95 (49.2)	32 (53.3)	0.082
**During Medical School, the Student has:**			
Authored a manuscript	8 (4.1)	4 (6.7)	0.112
Conducted research	65 (33.7)	26 (43.3)	0.196
Presented research findings	5 (2.6)	3 (5.0)	0.126
A physician-scientist role model	37 (19.2)	21 (35.0)	0.362
A research mentor	69 (35.8)	36 (60.0)	0.500
Participated in formal research coursework	12 (6.2)	4 (6.7)	0.018
**Proposed Mediators of Research Career Intention**			
Research Self-efficacy	33.84(5.65)	36.49(4.95)	-0.500
Outcome Expectations	3.28 (0.71)	3.60 (0.80)	0.434
**Medical Students are Offered:**			

© 2026 Hajduk AM et al. *JAMA Network Open*.

Financial support for student research	115 (59.6)	38 (63.3)	0.071
Summer research stipend	106 (54.9)	35 (58.3)	0.069
Full-Year research stipend	20 (10.4)	7 (11.7)	0.042
Financial support for research expenses	27 (14.0)	15 (25.0)	0.281
Funds to travel to a research meeting	53 (27.5)	24 (40.0)	0.268
Opportunities for full-time research	86 (44.6)	29 (48.3)	0.063
**Primary Reason for Conducting Research:**			0.545
Develop technical/transferable skills	9 (4.7)	4 (6.7)	
Satisfy intellectual curiosity	10 (5.2)	8 (13.3)	
Satisfy curricular/graduation requirements	12 (6.2)	1 (1.7)	
Increase competitiveness for residency	70 (36.3)	15 (25.0)	
Contribute to scientific advancements	5 (2.6)	3 (5.0)	
Enhance health equity	11 (5.7)	5 (8.3)	
Take a break from other clinical/academic duties	1 (0.5)	0 (0.0)	
Support career goal of being a researcher	0 (0.0)	2 (3.3)	
Other	74 (38.3)	22 (36.7)	
**The research scientist career path is presented:**			0.249
Somewhat negatively	10 (5.2)	3 (5.0)	
Neither	73 (37.8)	17 (28.3)	
Somewhat positively	56 (29.0)	23 (38.3)	
Very positively	41 (21.2)	15 (25.0)	
**Medical School Research Experience Taught How To:**			
Ask questions	36 (18.7)	20 (33.3)	0.353
Analyze data	49 (25.4)	20 (33.3)	0.192
Use new techniques	27 (14.0)	11 (18.3)	0.189
Develop methods	29 (15.0)	15 (25.0)	0.254
Critically review literature	39 (20.2)	18 (30.0)	0.230
Write a manuscript	24 (12.4)	7 (11.7)	0.227
Incorporate guidelines	24 (12.4)	10 (16.7)	0.190
**The student has a research mentor**			0.500
Yes	69 (35.8)	36 (60.0)	

© 2026 Hajduk AM et al. *JAMA Network Open*.

No	124 (64.2)	24 (40.0)	
**Student's Research Mentor is Skilled in:**			
Employing strategies to enhance research understanding			0.617
Yes	48 (24.9)	32 (53.3)	
No	17 (8.8)	3 (5.0)	
N/A	124 (64.2)	24 (40.0)	
Maintaining effective communication			0.601
Yes	56 (29.0)	33 (55.0)	
No	12 (6.2)	1 (1.7)	
N/A	124 (64.2)	24 (40.0)	
Addressing diversity			0.531
Yes	47 (24.4)	28 (46.7)	
No	18 (9.3)	7 (11.7)	
N/A	124 (64.2)	24 (40.0)	
Aligning expectations			0.588
Yes	55 (28.5)	34 (56.7)	
No	12 (6.2)	2 (3.3)	
N/A	124 (64.2)	24 (40.0)	
Fostering independence			0.499
Yes	55 (28.5)	30 (50.0)	
No	13 (6.7)	4 (6.7)	
N/A	124 (64.2)	24 (40.0)	
Promoting professional development			0.552
Yes	52 (26.9)	31 (51.7)	
No	14 (7.3)	3 (5.0)	
N/A	124 (64.2)	24 (40.0)	
Answering emails			0.587
Very Dissatisfied	0 (0.0)	1 (1.7)	
Dissatisfied	2 (1.0)	2 (3.3)	
Neutral	10 (5.2)	3 (5.0)	
Satisfied	26 (13.5)	10 (16.7)	

© 2026 Hajduk AM et al. *JAMA Network Open*.

Very Satisfied	29 (15.0)	20 (33.3)	
N/A	124 (64.2)	24 (40.0)	
Meeting regularly with mentee			0.637
Very Dissatisfied	0 (0.0)	1 (1.7)	
Dissatisfied	1 (0.5)	0 (0.0)	
Neutral	16 (8.3)	3 (5.0)	
Satisfied	24 (12.4)	17 (28.3)	
Very Satisfied	26 (13.5)	15 (25.0)	
N/A	124 (64.2)	24 (40.0)	
MCA Average			0.521
Yes	51 (26.4)	29 (48.3)	
No	10 (5.2)	3 (5.0)	
N/A	124 (64.2)	24 (40.0)	
**School Climate:**			
MSLES	3.81 (0.38)	3.83 (0.43)	-0.062
DES Score	4.11 (0.54)	4.08 (0.56)	0.044
Everyday Discrimination Scale	1.57 (0.63)	1.66 (0.75)	-0.136
Experience of Orientation-based Discrimination	8 (4.1)	2 (3.3)	0.043
Experience of Race-based Discrimination	19 (9.8)	8 (13.3)	0.109
Experience of Sex-based Discrimination	16 (8.3)	9 (15.0)	0.210
Mistreatment	33 (17.6)	10 (16.7)	0.025
Race Climate	5.81 (0.96)	5.83 (0.89)	-0.026
Institution is URiM supportive	160 (82.9)	49 (81.6)	0.336
Institution has URiM Leadership	165 (85.5)	53 (88.3)	0.084
Student reports sense of belonging as:			0.221
Low	4 (2.1)	2 (3.3)	
Some	16 (8.3)	2 (3.3)	
High	173 (89.6)	54 (90.0)	
**The student has limited time to engage in academic pursuits due to:**			
Outside activism	13 (6.7)	6 (10.0)	0.112
Outside committee	16 (8.3)	6 (10.0)	0.083

© 2026 Hajduk AM et al. *JAMA Network Open*.

Outside DEI work	10 (5.7)	5 (8.3)	0.096
Outside family	23 (11.9)	12 (20.0)	0.241
Outside employment	15 (7.8)	6 (10.0)	0.076
Outside volunteer	43 (22.3)	10 (16.7)	0.165

eReferences

1. Association of American Medical Colleges. Matriculating Student Questionnaire (MSQ) | Association of American Medical Colleges. *https://www.aamc.org/data-reports/students-residents/report/matriculating-student-questionnaire-msq*.

2. National Institutes of Health (NIH). Racial and Ethnic Categories and Definitions for NIH Diversity Programs and for Other Reporting Purposes. April 2015. https://grants.nih.gov/grants/guide/notice-files/not-od-15-089.html#:~:text=Diversity%20in%20NIH%20Programs,Data%20on%20Race%20and%20Ethnicity. Accessed September 29, 2025.

3. National Institute of Health. Disadvantaged background | ERA. *https://www.era.nih.gov/commons/disadvantaged_def.htm*.

© 2026 Hajduk AM et al. *JAMA Network Open*.

4. Association of American Medical Colleges. Association of American Medical Colleges Year Two Questionnaire (Y2Q). https://www.aamc.org/data-reports/students-residents/report/year-two-questionnaire-y2q. Accessed August 6, 2025.

5. International Association for the Evaluation of Educational Achievement. PIRLS 2016 Assessment Framework. https://timssandpirls.bc.edu/pirls2016/downloads/P16_Framework_2ndEd.pdf. Accessed November 19, 2025.

6. Ha TC, Ng S, Chen C, et al. Inclination towards research and the pursuit of a research career among medical students: an international cohort study. *BMC Med Educ*. 2018;18(1):86. doi:10.1186/s12909-018-1202-6.

7. Saha S, Beach MC, Cooper LA. Patient Centeredness, Cultural Competence and Healthcare Quality. *J Natl Med Assoc*. 2008;100(11):1275-1285. doi:10.1016/S0027-9684(15)31505-4.

8. Jeffe DB, Andriole DA. Prevalence and predictors of US medical graduates' federal F32, mentored-K, and R01 awards: a national cohort study. *J Investig Med*. 2018;66(2):340-350. doi:10.1136/jim-2017-000515.

9. Fleming M, House S, Hanson VS, et al. The Mentoring Competency Assessment. *Academic Medicine*. 2013;88(7):1002-1008. doi:10.1097/ACM.0b013e318295e298.

10. Berk RA, Berg J, Mortimer R, Walton-Moss B, Yeo TP. Measuring the Effectiveness of Faculty Mentoring Relationships. *Academic Medicine*. 2005;80(1):66-71. doi:10.1097/00001888-200501000-00017.

11. Sozio SM, Chan KS, Beach MC. Development and validation of the Medical Student Scholar-Ideal Mentor Scale (MSS-IMS). *BMC Med Educ*. 2017;17(1):132. doi:10.1186/s12909-017-0969-1.

12. Chemers MM, Zurbriggen EL, Syed M, Goza BK, Bearman S. The Role of Efficacy and Identity in Science Career Commitment Among Underrepresented Minority Students. *Journal of Social Issues*. 2011;67(3):469-491. doi:10.1111/j.1540-4560.2011.01710.x.

13. Estrada M, Woodcock A, Hernandez PR, Schultz PW. Toward a model of social influence that explains minority student integration into the scientific community. *J Educ Psychol*. 2011;103(1):206-222. doi:10.1037/a0020743.

© 2026 Hajduk AM et al. *JAMA Network Open*.

14. Marshall RE. Measuring the medical school learning environment. *Academic Medicine*. 1978;53(2):98-104. doi:10.1097/00001888-197802000-00003.

15. Person SD, Jordan CG, Allison JJ, et al. Measuring Diversity and Inclusion in Academic Medicine. *Academic Medicine*. 2015;90(12):1675-1683. doi:10.1097/ACM.0000000000000921.

16. Reid LD, Radhakrishnan P. Race matters: The relation between race and general campus climate. *Cultur Divers Ethnic Minor Psychol*. 2003;9(3):263-275. doi:10.1037/1099-9809.9.3.263.

17. Williams DR, Yan Yu, Jackson JS, Anderson NB. Racial Differences in Physical and Mental Health. *J Health Psychol*. 1997;2(3):335-351. doi:10.1177/135910539700200305.

18. Association of American Medical Colleges. Graduation Questionnaire (GQ) | Association of American Medical Colleges. *https://www.aamc.org/data-reports/students-residents/report/graduation-questionnaire-gq*.

19. Kroenke K, Spitzer RL, Williams JBW, Lowe B. An Ultra-Brief Screening Scale for Anxiety and Depression: The PHQ-4. *Psychosomatics*. 2009;50(6):613-621. doi:10.1176/appi.psy.50.6.613.

20. Duckworth AL, Peterson C, Matthews MD, Kelly DR. Grit: Perseverance and passion for long-term goals. *J Pers Soc Psychol*. 2007;92(6):1087-1101. doi:10.1037/0022-3514.92.6.1087.

21. West CP, Dyrbye LN, Sloan JA, Shanafelt TD. Single Item Measures of Emotional Exhaustion and Depersonalization Are Useful for Assessing Burnout in Medical Professionals. *J Gen Intern Med*. 2009;24(12):1318-1321. doi:10.1007/s11606-009-1129-z.

22. Wang B, Andrews W, Bechtoldt MN, Rohrmann S, de Vries RE. Validation of the Short Clance Impostor Phenomenon Scale (CIPS-10). *European Journal of Psychological Assessment*. 2024;40(2):158-168. doi:10.1027/1015-5759/a000747.

23. Black ML, Curran MC, Golshan S, et al. Summer Research Training for Medical Students: Impact on Research Self-Efficacy. *Clin Transl Sci*. 2013;6(6):487-489. doi:10.1111/cts.12062.

© 2026 Hajduk AM et al. *JAMA Network Open*.

24. Byars-Winston A, Rogers J, Branchaw J, Pribbenow C, Hanke R, Pfund C. New Measures Assessing Predictors of Academic Persistence for Historically Underrepresented Racial/Ethnic Undergraduates in Science. *CBE—Life Sciences Education*. 2016;15(3):ar32. doi:10.1187/cbe.16-01-0030.

25. U.S. Department of Justice Civil Rights Division. Americans with Disabilities Act of 1990, As Amended. *2008*. https://www.ada.gov/law-and-regs/ada/. Accessed September 24, 2025.

© 2026 Hajduk AM et al. *JAMA Network Open*.
