# Supplementary material for: Longitudinal Evaluation of Research Career Intentions Among US Medical Students
Source: JAMA Netw Open. 2026 May 12;9(5):e2611430. doi: 10.1001/jamanetworkopen.2026.11430 (PMC13169399; doi:10.1001/jamanetworkopen.2026.11430)
Supplement: Supplement 2. — Data Sharing Statement [file jamanetwopen-e2611430-s002.pdf]

Data Sharing Statement

Hajduk. Research Career Intentions Among Medical Students. *JAMA Netw Open*. Published May 11, 2026. doi:10.1001/jamanetworkopen.2026.11430

Data
Data available: No

Additional Information
Explanation for why data not available: The data used in this report is a part of an ongoing longitudinal study and will be made available upon completion of the study.
